# Supplementary material for: Accurate mapping of mitochondrial DNA deletions and duplications using deep sequencing
Source: PLoS Genet. 2020 Dec 14;16(12):e1009242. doi: 10.1371/journal.pgen.1009242 (PMC7769605; doi:10.1371/journal.pgen.1009242)
Supplement: Attachment — Submitted filename: Point by point response.pdf [file pgen.1009242.s005.pdf]

Point-by-point response
Manuscript PGENETICS-D-20-00780

"Accurate mapping of mitochondrial DNA deletions and duplications using deep sequencing"

Dear reviewers,

We wish to thank you for your insightful comments. Based on your feedback, we have made several considerable improvements, including more comprehensive benchmarking based on new extensive simulations, application to ICGC cancer whole genome sequencing data, and refinements that improve the reports generated by our tool.

For clarity, the reviewer's remarks are shown below in black while our responses are in blue.

Reviewer #1: The authors describe a method for identification of structural variation in mitochondrial genomes. A selling point of the method is its ability to accurately classify SVs as deletions or duplications, in addition to its ability to do so from whole genome, whole exome or transcriptome sequencing data. The authors demonstrate the accuracy of their method on simulated data and the utility when applied to mouse models of mitochondrial disorder.

The paper is clearly and the figure quality is good. I have the following major concerns.

1. As the authors have noted, discerning duplications from deletions is unidentifiable for a circular genome without additional information. Using the two replication origins is reasonable, but depending on the location of the deletion / duplication, there may still be ambiguity. The authors should mention this ambiguity and describe any rules they apply to decide between duplications and deletions in this situation.

There is an inherent ambiguity when using short read sequencing: for example, any potential small deletion may in principle also represent a large complementary arc duplication. However, assuming that both origins need to be retained, there is (somewhat surprisingly) no ambiguity.

Implicit in the way our algorithm is designed and described (although not spelled out in this way), is this logic: 1) origins should preferably be completely unaltered, and if this is not possible 2) no origin should ever be deleted.

A deletion in the minor or major arc can thus only be a deletion (the alternative is a duplication of the complementary arc that would also duplicate the origins). A duplication in the minor or major arc can only be a duplication (the alternative is a complementary deletion that would remove both origins). A deletion of an origin should rather be a duplication of the complementary arc (which would duplicate the other origin but ensure none are deleted).

To further clarify this to the reader, we have added the following sentence to Results (row 144):

"The preferred interpretation is thus one where both origins are unaltered or, when this is not possible, none are deleted. The deletion/duplication classification is always non-ambiguous, since only one interpretation will satisfy these criteria while the other will violate them."

2. The simulations as described are insufficient to fully evaluate Mitosalt or competing methods. Unless I misunderstand, the authors simulated dataset included only 6 events. By contrast, MitoMut was benchmarked on a simulated dataset described as follows. “We simulated 3000 paired-end Illumina whole-genome sequencing experiments with one deletion each. Of the simulations, 1000 had small deletions (5-30 bps), 1000 had medium deletions (31-500 bps), and 1000 had large deletions (500-5000 bps).” The authors should benchmark on a more comprehensive dataset, ideally one with similar scale to that described in the MitoMut paper.

We agree this is relevant, and more comprehensive simulations and evaluations have now been made. Specifically, we simulated WGS data containing low heteroplasmy level (0.5%) deletions and duplications of various sizes (50, 500, 2000 nt). Each dataset contained 200 different events of a single type distributed across the major and minor arcs. Additionally, 500 nt deletions with 5 nt non-template insertions were similarly evaluated, to satisfy a comment from reviewer 2. A wild-type dataset was also generated using a non-altered reference genome, to enable assessment of false positives. Notably, in contrast to many other studies, complete WGS datasets were simulated including nuclear reads: this considerably increases the amount of data generated but better mimics the real-life situation. The results of our evaluations are presented in Fig. 2.

Briefly, 100% of the events were detected, far outperforming the majority of tools. The only contender was Zambelli et al., which had similar sensitivity but lower precision when it came to exact determination of breakpoint coordinates (the script is also not very user friendly). MitoSAlt also correctly classified all events as deletions or duplications (this assessment is missing entirely in the other tools). We want to thank the reviewer for pushing us to make this effort.

3. In the simulation results, no mention is made of the number of false positives produced by mitosalt or the other tools. Mitosalt appears to be more sensitive but how specific is it relative to other methods.

Reassuringly, none of the tools including ours detected false positives in the wild type data, and this is now mentioned in Results (row 233).

4. The authors have applied their method to WGS and MT enriched WGS sequencing but have not provided any evidence supporting their claim that the method works on whole exome or transcriptome sequencing.

This is a valid point: Our tool was designed and evaluated with whole genome sequencing (mtDNA enriched or not) in mind, since this is clearly the most suitable type of data. Since the same principle should be applicable to RNA-seq and WXS, we mentioned these possibilities, although in practice there are limitations, e.g. WXS typically displays limited mtDNA coverage. To not cause confusion, we have now changed the abstract to mention only genomic sequencing data. We also added a sentence to discussion mentioning that the tool is primarily designed to be used on WGS or mtDNA enriched WGS data although the same workflow should in principle also be applicable to RNA-seq or WXS.

Reviewer #2: In this manuscript entitled "accurate mapping of mitochondrial DNA deletions and duplications using deep sequencing", the authors generated a straightforward tool, or MitoSAlt, to call the mitochondrial structural variations. Structural variations in mitochondrial DNA have not been extensively studied due to technical difficulties. The authors compared their tool with a few publicly available tools, such as MitoDel, Splice-Break, EKLIPse, MitoMut and a Perl script by Zambelli. From the benchmark study, the authors concluded that the performance of MitoSAlt is superior to these tools. I feel that MitoSAlt is very useful

and will be used in future mitochondrial genome studies. The manuscript also reads well. With a few additional validations, I think the manuscript is suitable for publication in Plos Genetics.

Minor comments:
(1) Sensitivity: what is the sensitivity of mtDNA structural variation detection of MitoSAlt? I believe that it depends on the mtDNA sequence read-depth and some features of mtDNA structural variants. However, I am still wondering the minimum heteroplasmy of mtDNA variants that can be detected by MitoSAlt in given read depth. Is it able to show any metrics to the authors?

The initial simulations were based on high heteroplasmy (16.7%) events only, which indeed does not say anything about the sensitivity for low heteroplasmy events. We have now added new simulations results, where sensitivity was tested on more than 1,000 deletions and duplications present at 0.5% heteroplasmy (Fig. 2). 100% of these events were detected, despite the size of the simulated data (50,000,000 reads) being only about 1/20th of our patient WGS datasets (Fig. 4). The exact lower limit will be unique to each case, as it is a function of read depth as well as mitochondrial copy number. At least we can confidently say that events at 0.5% heteroplasmy can be easily detected even when read depth is low or moderate. A comment about this was added to the discussion.

(2) In structural variations, sometimes non-template nucleotide insertions are engaged in the breakpoints. How these sequences are handled in MitoSAlt?

The common assumption seems to be that, in contrast to the nuclear genome where NHEJ may introduce non-template sequences, this may not be so common in mitochondria. However, to ensure that our tool can nevertheless handle this situation, we have now tested MitoSAlt on a large number of simulated 500 bp deletions harbouring 5 bp random non-template insertions at the breakpoint (Fig. 2b, “500+5nt”). Reassuringly, these were detected at 100% sensitivity (200/200), despite being present at low (0.5%) heteroplasmy. We thank the reviewer for bringing this to our attention.

(3) I am wondering how breakpoint sequence microhomology is treated in MitoSAlt calls.

To more easily enable the user to assess microhomology at breakpoints, we have now added three new output columns to the final result file that indicate 1) sequence around the first breakpoint 2) sequence around the second breakpoint and 3) the longest direct repeat sequence in between the two breakpoints (spanning or flanking both breakpoints).

(4) Is there any possibility of false-positives due to hidden NUMTs? For example, if a NUMT sequence is equivalent to a mitochondrial sequence with a large deletion, and the MUMT is not represented in the reference genome, then the sequence will be misaligned to the mitochondrial reference genome and may appear as a mitochondrial DNA structural variation at ~1% heteroplasmic level.

We fully agree in principle, and this may be particularly relevant in mouse. In practice, there are essentially no detections at all in the wild type mouse controls presented in the lower panel of Fig. 4b. In comparison, the mutant mice show extensive alterations. We have now also added simulated data generated from a wild type human genome that included both nuclear and mitochondrial reads (row 233). No false positives were detected in this data. Thus, although the reviewer makes a good point, we believe that in practice this does not seem to be causing false positive detections.

(5) Figure 3. I am wondering whether the authors can further validate the variations identified by MitoSAlt with another technique.

The reviewer brings up a relevant point. However, a major challenge is the low heteroplasmy levels in the mice models. This may represent precisely the type of situation where NGS-based detection outshines traditional methods, which will struggle to find a signal.

In Fig. 2 (Fig. 3 in the new version of the manuscript), we validated our pipeline using human samples with a single type of deletion or duplication present at high levels of heteroplasmy. For these samples, the alterations are readily detectable using long-range PCR, allowing us to clearly verify that our pipeline correctly detects and classifies duplication and deletion patterns.

In the mouse samples, the combined levels of duplications reach a few percent of total mtDNA at most. Further complicating the issue, rather than single events there is a distribution of different sizes. This smears the signal and further reduces its visibility alongside the wildtype band. We have not been able to detect these events using long range PCR of the *Twkn* and *Mgme1* mutant mice (no more material was available for *Polg*), and we believe this is the reason.

It should be noted that similar breakpoint positions as we here identify in the *Mgme1*$^{-/-}$ mouse have been detected by single-molecule amplification and subsequent sequencing of muscle mtDNA from patients with loss-of-function mutations in human *MGME1* (Nicholls et al, Hum Mol Genet. 2014 Dec 1;23(23):6147-62). This gives further support for our observations, and reference was inserted into the discussion to underscore this. In addition, others have studied the *Polg*^{D257A} mouse and have suggested duplications in the same region based on short amplicon PCR, although they were not successful using long-range PCR (Williams et al, Cell Metab. 2010 Dec 1;12(6):675-82). While PCR using short breakpoint-spanning amplicons does not prove the deletion/duplication nature of the event, a deletion appears unlikely as it would imply that most of mtDNA is lost with only a very small circular molecule remaining. A reference was added to the discussion to highlight this study.

In summary, although difficult to verify with long-range PCR, we feel there is good reason to believe results are accurate (the new simulations now added to Fig. 2 of the manuscript, where MitoSAlt correctly identifies and classifies a large number of events without false positive detections, further strengthens that the specificity of the method is high).

(6) How precise the heteroplasmic level estimates of variant mtDNA?

We have now added new extensive simulations that include 7 datasets, each harboring 200 deletions and duplications, presented in Fig. 2. These events were all present at 0.5%, to be compared with an average estimated heteroplasmy level in each dataset between 0.38% and 0.57%. Estimates should be less noisy as heteroplasmy levels or sequencing depth increases, due to reduced sampling errors. It should be noted that heteroplasmy levels can be expected to be somewhat underestimated: when a breakpoint is too far to the edge of a read, it will not produce a valid split alignment and will therefore drop out. We have not attempted to correct for this effect (which is not entirely trivial as read lengths and hence dropout rates can vary) as we feel that estimates should be accurate enough for most applications.

(7) In a recent paper (Yuan Yuan et al., Nature Genetics 2020, https://www.nature.com/articles/s41588-019-0557-x), the authors identified mtDNA somatic structural variations in three human cancer genomes. The authors may want to test MitoSAlt to show the performance of their tool.

We have now applied for access and subsequently downloaded these WGS datasets. Reassuringly, the alterations identified based on read depth in the cited paper are precisely confirmed by our method. These results are presented in Supplementary Fig. 2.
